# Do Commercial Insect Repellents Provide Protection against the Tick *Amblyomma sculptum* (Acari: Ixodidae)?

**DOI:** 10.3390/pathogens13010009

**Published:** 2023-12-21

**Authors:** Beatriz Rodrigues de Barcelos, Nathália Gabriela Silva Santos Coelho, Mayara Macedo Barrozo Santos, Francisca Letícia Vale, Ana Lúcia Coutinho Teixeira, Lainny Martins Pereira e Souza, Viviane Zeringóta, Caio Márcio de Oliveira Monteiro, Chesterton Ulysses Orlando Eugenio, Marcos Takashi Obara

**Affiliations:** 1 Postgraduate Program in Health Sciences and Technologies, University of Brasilia, Metropolitan Center, Conjunto A, Lot 01, Federal District, Brasilia 72220-275, Brazil; 2 Department of Biological Sciences, Catholic University of Brasilia, QS 07, Lot 01, Taguatinga, Federal District, Brasilia 71966-700, Brazil; nathaliagsilvasc@gmail.com; 3 Postgraduate Program in Animal Science, Federal University of Goiás, Nova Veneza, km 8, Samambaia Campus, Goiania 74001-970, Brazil; may_macedo_@hotmail.com (M.M.B.S.); leticiadetecta@gmail.com (F.L.V.); luciacoutinho13@gmail.com (A.L.C.T.); lainnyjordana@gmail.com (L.M.P.e.S.); caiosat@gmail.com (C.M.d.O.M.); 4 Institute of Tropical Pathology and Public Health, Federal University of Goias, Goiania 74605-220, Brazil; viviane_zeringota@ufg.br; 5 Instituto Federal de Educação, Ciência e Tecnologia de Goiás, São Bartolomeu Street, Luziania 72811-580, Brazil; chesterton.ucb@gmail.com

**Keywords:** *Amblyomma cajennense* complex, *Amblyomma sculptum*, resistance, Brazilian Spotted Fever, DEET, Icaridin and IR3535

## Abstract

*Amblyomma sculptum* is a species of public health interest because it is associated with the transmission of the bacteria that causes Brazilian Spotted Fever (BSF). The use of repellents on humans is a prophylactic measure widely used to provide protection against a series of arthropod vectors, including mosquitoes and ticks. However, in Brazil, the effectiveness of commercial repellents against *A. sculptum* is little known. Therefore, it is necessary to carry out specific studies to evaluate the repellency of these commercial products, registered for use against mosquitoes, against the star tick. The main goal of the present work was to evaluate the effectiveness of six commercial repellents against *A. sculptum*. Unfed nymphs, aged between two and eight weeks, were tested against products based on DEET (OFF!^®^ and Repelex^®^), Icaridin (Exposis^®^ and SBP^®^), and IR3535 (Johnsons and Henlau). Three bioassays were conducted to evaluate tick behavior: (i) filter paper, (ii) fingertip, and (iii) field. All bases tested showed high repellent activity, differing significantly (*p* < 0.05) from the control. It was observed the formulation with DEET resulted in the best results in the filter paper bioassay. In the fingertip bioassay, the DEET base repelled a greater number of ticks compared to Icaridin. In the field bioassay, there was no significant difference between the Icaridin base and DEET, and both formulations differed from the control (*p* < 0.05). The six formulations tested showed significant percentages of repellency against ticks; however, for the fingertip and field bioassays, the products OFF!^®^, Repelex^®^, and Exposis^®^ were tested as they showed better performance in the filter paper test. OFF!^®^ showed the best percentage of repellency (100%), followed by Repelex^®^ (96.8%), and Exposis^®^ (93.1%), considering the two-hour period of the bioassay-field-test. Proving the effectiveness of repellents on the market against *A. sculptum* presented in this study is crucial, since this is the main ectoparasite of humans that can transmit *Rickettsia rickettsii* when infected. The effectiveness of commercial insect repellents against other tick species that parasitize humans can also be explored.

## 1. Introduction

Ticks (Acari: Ixodida) are ectoparasitic arthropods that have a wide distribution, occupying all six zoogeographic regions, mainly in the tropics [1]. Currently, more than 900 species are known globally, with around 70 found in Brazil. Among ticks, the genus *Amblyomma* has great medical and veterinary importance and wide geographic distribution in tropical and subtropical regions, with around 32 species found in Brazil [2].

Within the genus *Amblyomma*, the species *Amblyomma sculptum* stands out, belonging to the *A. cajennense* complex, distributed in Argentina, Bolivia, Paraguay, and Brazil [3]. In Brazil, *A. sculptum* is found in all regions of Brazil, being registered in several states [4]. In these areas, this species is associated with the transmission of infectious agents [5] and is the main vector of the bacterium *Rickettsia rickettsii*—the etiological agent of Brazilian Spotted Fever (BSF) [6].

Since 2001, BSF has been a zoonosis that must be notified to local health authorities, and epidemiological investigation must be initiated within 48 h. The investigation form feeds into the Ministry of Health’s Notifiable Diseases Information System (SINAN-MS) as an official notification instrument. The importance of notification is also due to the possibility of an outbreak, requiring the adoption of immediate control measures [7].

According to the Epidemiological Bulletin of the Ministry of Health [8], between 2007 and 2021, 36,497 cases of Spotted Fever were reported in Brazil, of which 2545 cases were confirmed. However, most cases are underreported [9]. Most of the BSF records occur in urban areas, with the Southeast region having the highest incidence rate of cases, due to the presence of *A. sculptum* and amplifying hosts [10,11].

The epidemiological scenario of BSF in the three Brazilian states with the highest lethality coefficient (São Paulo, Rio de Janeiro, and Minas Gerais) demonstrates a strong tendency for cases to increase, especially in the months of August to November, when there are higher incidences of nymphs. Capybaras and opossums play a relevant role in the BSF epidemiological chain, as they amplify *rickettsiae*, in addition to being food and transport for potentially infected ticks [12].

The World Health Organization (WHO) recommends the use of protection mechanisms in endemic areas, including the use of repellents [13]. The development of synthetic repellents began around 1930 in the United States and United Kingdom. This process intensified with the Second World War to ensure the protection of soldiers against the bites of arthropod vectors [14].

Repellents are important prophylactic tools for people living or traveling to endemic areas for vector-borne diseases. Initially, repellents were developed from plant extracts, formulated with a single active ingredient or repellent substance, which were burned to produce smoke. Molecularly, these products evolved and started to contain active substances such as DEET, which cause a maladjustment in the olfactory system of medically important arthropods [15]. Thus, repellents are chemical substances that produce a loss of response to the host, that is, they produce locomotors stimulus away from the food source, altering the arthropod’s behavior [16].

Repellents are regulated as cosmetics; however, in the technical requirements for granting registration, there are specifications only for insect repellents, according to RDC (Collegiate Board Resolution) n°. 19, of 10 April 2013 [17], and there is no regulation for ticks. Hence, given the lack of regulation on specific registration for ticks and the need to prevent BSF, it is important to evaluate the efficiency of commercial repellents used, mainly against mosquitoes on *A. sculptum* in order to contribute to the development of public policies that help prevent BSF.

In this sense, the objective of this study was to evaluate the chemical repellency of six commercial products based on DEET, IR3535, and Icaridin against *A. sculptum*, to improve BSF prevention and control actions.

## 2. Materials and Methods

### 2.1. Tick Colony

To carry out the experiments in the laboratory, *A. sculptum* nymphs were used, provided by the Ixodology Laboratory (Labix) of the Faculty of Veterinary Medicine of the Federal University of Uberlandia (FAMEV/UFU). The ticks were fed on rabbits (*Oryctolagus cuniculus*), and this colony was started in 2019 with specimens collected at Serra Farm, located in the city of Araguapaz, Goiania [18].

The maintenance of ticks was approved by the Ethics Committee on the Use of Animals (CEUA) of the Federal University of Uberlandia, according to protocol n° 069/18. Nymphs were sent to Brasilia-DF, where they were placed in desiccators kept in BOD-type greenhouses at 20 °C and 85% relative air humidity for a maximum period of 60 days, and taken to the Laboratory of Biology, Ecology and Tick Control (LABEC) from the Federal University of Goias (UFG) to carry out the experiments.

### 2.2. Repellents

The repellency tests were conducted at LABEC, located at the Veterinary Parasitology Center (CPV) of the UFG Veterinary School, in the period between 2019 and 2021.

To carry out the repellency tests, six commercial liquid repellents registered with ANVISA were used, based on DEET, Icaridin, and IR3535, as shown in Table 1.

### 2.3. Repellency of Bioassays

**Figure 1:**
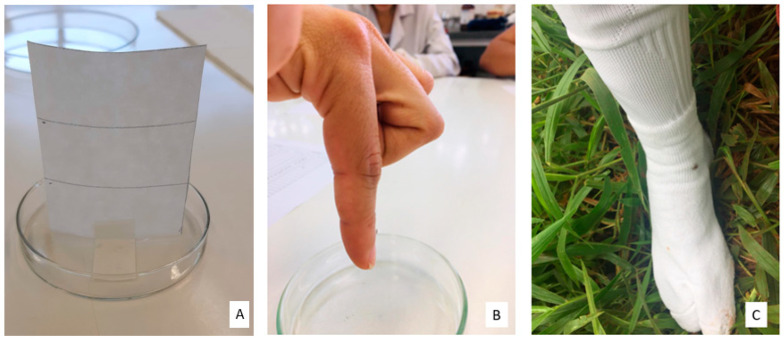
Behavioral assessment bioassays with *A. sculptum.* (**A**) Filter paper bioassay (rectangular paper filter measuring 6 cm × 10 cm and glass petri dish measuring 9 cm × 1.5 cm), (**B**) Fingertip bioassay (index finger) and, (**C**) Field bioassay in an area infested with *A. sculptum*.

Unfed nymphs, aged two weeks to two months, were used, as established by Soares et al. [19]. Two laboratory bioassays were carried out to evaluate the repellency of the *A. sculptum* tick: (i) filter paper bioassay and (ii) fingertip bioassay. Laboratory bioassays were carried out at a temperature of 27 °C and humidity of 70%. Figure 1 shows images of the bioassays carried out in this study. Subsequently, a field bioassay was carried out at the facilities of the School Farm of Federal University of Goiás (UFG), during the period of nymph infestation, which occurred in July 2021.

### 2.4. Filter Paper Bioassay

For the in vitro evaluation of the activity of the six repellents, a rectangular filter paper (6 cm × 10 cm) divided into three bands was used, with the intermediate band (3 cm × 6 cm) being treated with 90 µL of the repellent, followed by the methodology proposed by Carrol et al. [20], with adaptations. In the control group, 99.5% ethyl alcohol was used. The rectangle was placed on a 100 mm diameter glass Petri dish and fixed with adhesive tape (Figure 1A).

After a 10-min interval, waiting for the solvent to evaporate, five nymphs were placed on the lower region of the paper and the ticks’ movement was observed for 1 min. This assessment was repeated every hour, in a total of five hours of testing. Eight replications were performed for each commercial formulation, as well as for the control. It should be noted 240 nymphs were used for each repellent tested, that is, five nymphs per repetition and 40 nymphs per repellent in each time interval tested. Nymphs were discarded after each observation.

Ticks that, after the end of the time interval, remained on the untreated portion of the paper, those that fell into the Petri dish and those that, in an upward movement, reversed direction when coming into contact with the treated area, were classified as repelled. Ticks that remained in the treated area or passed through and remained on the top of the paper were considered not repelled. With the results of the filter paper bioassay, the three best repellents were selected to perform the fingertip and field bioassay.

### 2.5. Fingertip Bioassay

For the fingertip repellency test, the methodology recommended by Schreck et al. [21], was applied, using the three repellents that showed the best results in the filter paper bioassay (OFF!^®^, Repelex^®^, and Exposis^®^). The repellent was applied to the proximal phalanx of the right index finger of each volunteer, while the control (99% ethanol) was used on the proximal phalanx of the left index finger. A total of five volunteers participated in the experiment.

The amount of substance used was 2.75 μL/cm^2^, following the methodology described by Carroll et al., with the solution being applied with a micrometric pipette. To calculate the treatment application area, the formula πdh was used, in which πd represents the length of the circumference of each volunteer’s index finger, and h corresponds to the distance between the second and third skin folds of the finger. Thus, each volunteer had a specific amount of substance applied according to the area calculation. After application, a 10-min interval was waited for the solvent to evaporate and the test began.

The volunteers positioned their index finger horizontally, and then a nymph was placed on the first phalanx of the index finger. Subsequently, the finger was positioned vertically, with the tip down (Figure 1B). Due to the negative geotropism of *A. sculptum* nymphs, the expected behavior was they would climb up the finger if they were not repelled.

From the moment the tick started its upward movement, one minute was recorded. Ticks that after the end of this time remained on the untreated portion of the finger, those that fell, and those that in an upward movement reversed direction when coming into contact with the treated area were considered repelled. For each repellent tested, 180 nymphs were used, with 6 nymphs used for each volunteer in each time interval tested, totaling 30 nymphs per repellent in each test period. All ticks were first tested on the control substance. Those that did not move vertically were discarded, and only those that crossed the area treated with the control were used.

### 2.6. Field Bioassay

In the field bioassay, an area known to be infested by *A. sculptum*, close to places where cattle and capybaras circulate at the School of Veterinary and Animal Science at the Federal University of Goiás, was selected. The methodology of Bissinger et al. [22] and Ogawa et al. [23] was adapted, using the three repellents that demonstrated the best performance in the first bioassay (OFF!^®^, Repelex^®^, and Exposis^®^).

The volunteers wore the appropriate PPE for the activity (white jumpsuit properly sealed with adhesive tape at the ends of the sleeves, with zippers or buttons). Each volunteer received 2 previously treated long socks (10 min before the beginning of the experiment, to evaporate the solvent). In each sock, 1 mL was applied for every 600 cm^2^ of treatment area, following a volume similar to that used in the filter paper methodology. The application always occurred in the mid-calf region, above the calf.

The 2-h experiment period was adopted, as recommended by Pajuaba-Neto [24] for the test in the environment, with dry ice and drag test. Each volunteer received different treatments, including OFF!^®^, Repelex^®^, Exposis^®^, and control (99% alcohol), with nine repetitions of each treatment performed.

The volunteers were instructed to walk randomly through an area measuring about 400 m^2^ at a slow pace, taking approximately 30 steps per minute, for a period of 15 min. Each substance was tested in different ways. Ticks were collected with transparent adhesive tape at 20, 40, 60, and 120 min, stored in properly identified and sealed plastic bags. During the test period, all ticks that crossed the calf area were collected and stored in plastic bags and were considered non-repellent. Those that did not pull up their socks and remained in the foot area were considered repelled and were not counted.

After completion of the test, the plastic bags containing the ticks classified as non-repellent were taken to LABEC, where they were stored in a freezer with at least −4 °C. Subsequently, the number of ticks was recorded and they were morphologically identified according to Martins et al. [25] and Martins et al. [26]. It is important to note all nymphs collected belong to the species *Amblyomma sculptum*.

The following formula is used to calculate the repellency: Repellency (%) = (C − T)/C × 100, where C represents the number of ticks not repelled in the control group and T represents the number of ticks not repelled in the treatment group.

All bioassays were authorized by the Animal Research Ethics Committee (CEP) of the Federal University of Goiás under registration no. 4.955.565.

### 2.7. Statistical Analysis

To compare the proportions of repelled and non-repelled ticks between the different substances tested in the bioassays (filter paper, fingertip, and field), statistical analyzes were performed using R (Version 4.1.2) [27]. The graphs were generated using the statistical program PAST v 4.0 [28]. The statistical analysis used was the Kruskal-Wallis test, followed by Dunn’s post-test for multiple pairwise comparisons (kruskal_test and dunn_test functions from the Rstatix package). The Kruskal-Wallis was performed for each bioassay (filter paper, fingertip, and field), and the Dunn’s test was used to perform pairwise comparisons between the repellents. For all statistical tests, *p* less than 0.05 was used as the statistical significance threshold.

## 3. Results

### 3.1. Laboratory Bioassays

The results of the filter paper bioassay with the selected repellents and the control substance are presented in Table 2. In this table it is possible to observe the order of repellency of the products was OFF!^®^ > Repelex^®^ > Exposis^®^ > SBP^®^ > Johnson^®^ > Henlau^®^.

The analysis demonstrated the repellents presented different repellency rates than the control group (Kruskal-Wallis test, *p* < 0.05). DEET-based repellents showed the best results (OFF!^®^—97.08% and Repelex^®^—95.42%) compared to those based on Icaridin (SBP^®^—93.33% and Exposis^®^ 94.58) and IR3535 (Henlau^®^—75.42% and Johnson^®^—76.25%). Furthermore, no significant difference was observed between Icaridin-based repellents (SBP^®^ and Exposis^®^) and DEET (OFF!^®^ and Repelex^®^); however, a difference was observed between IR3535 (Henlau^®^ and Jonhson^®^) and DEET, and IR3535 and Icaridin (Table 2).

The commercial repellents OFF!^®^, REPELEX^®^, and EXPOSIS^®^ were selected to carry out the fingertip bioassay because they showed the highest mean in repellency of *A. sculptum*.

The results of the fingertip bioassay with the commercial repellents OFF!^®^, Repelex^®^, and Exposis^®^ are presented in Table 3. The experiments used a total of five volunteers and there was no statistically significant difference in repellency between volunteers. The OFF!^®^ brand repellent repelled a greater number of ticks (Kruskal-Wallis test, *p* < 0.05) when compared to the repellents Repelex^®^ and Exposis^®^, with the latter two not differing from each other. It was verified the *A. sculptum* nymphs were strongly repelled by OFF!^®^ repellent, throughout the five hours of testing, with the percentage of repellency at 96.67%, against 47.7% for Repelex^®^, and 55.5% of Exposis^®^.

**Figure 2:**
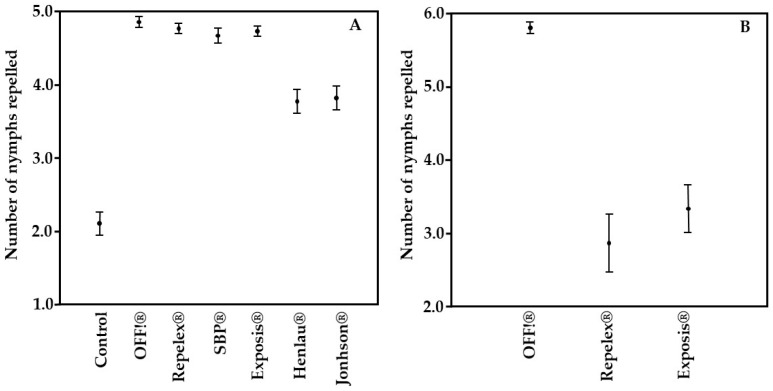
Mean number of *Amblyomma sculptum* nymphs exposed to different commercial repellents. (**A**) Filter paper bioassays, 2019. (**B**) Fingertip bioassay, 2020.

Figure 2A shows the mean number of nymphs repelled by the tested products, indicating a lower performance of Henlau^®^ and Jonhson^®^. In Figure 2B, it is possible to see OFF!^®^ presented the best result in the average number of nymphs repelled.

### 3.2. Field Bioassay

For the field bioassay, the 3 (three) repellents that presented the best results in laboratory bioassays (filter paper and fingertip) were used, namely: OFF!^®^ (DEET), Repelex^®^ (DEET), and Exposis^®^ (Icaridin). The field bioassay was carried out by four volunteers and there was no difference in repellency efficiency between them.

In the field bioassay, no significant difference was observed between the repellents Repelex^®^, Exposis^®^, and OFF!^®^ (Kruskal-Wallis test, *p* ≥ 0.05) (Table 4). All repellents tested demonstrated high percentages of repellency against *A. sculptum*. OFF!^®^ repellent showed the best percentage of repellency (100%), followed by Repelex^®^ (96.8%), and Exposis^®^ (93.1%), over the two hours of the test (Table 4).

**Figure 3:**
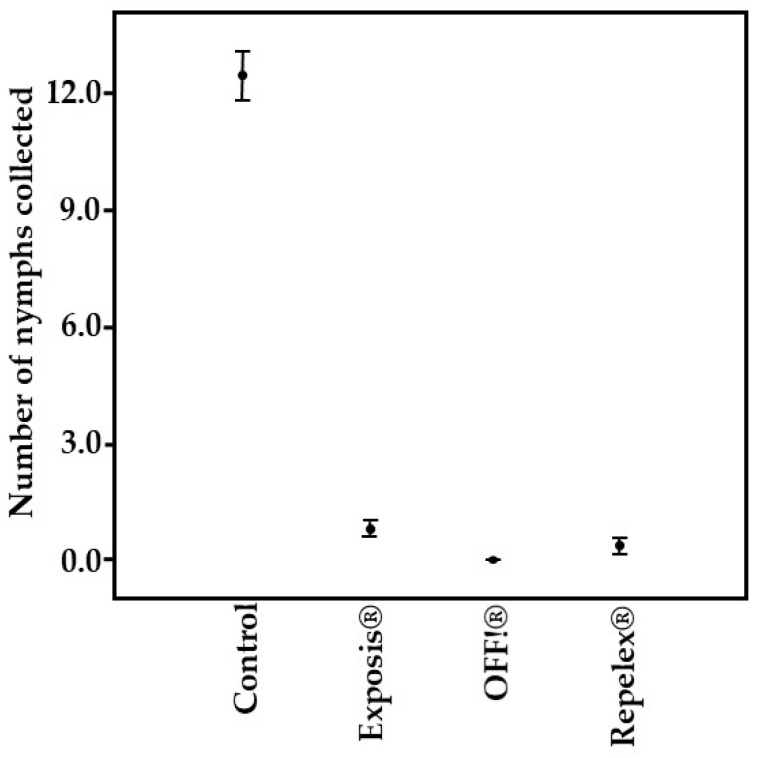
Mean number of *Amblyomma sculptum* nymphs captured in the field bioassay using different brands of repellents, conducted at the School of Agronomy at the Federal University of Goiás, Goiânia/GO, in June 2021.

It is observed statistically the repellents obtained similar efficiencies, with OFF!^®^ repellent having the best result (Figure 3), since it was the product in which no nymphs were captured during the bioassay period.

## 4. Discussion

The preventive measures against spotted fever, suggested by the Brazilian Ministry of Health, include the use of pants, boots, and shorts with long sleeves. In addition, it recommends the use of repellents, but there is no recommendation about which product to use. In this way, the present study allows, for the first time, the recommendation of the use of repellent products against the star tick, to improve the measures of prevention and control of Brazilian spotted fever.

The results obtained in this research supported the use of commercial repellents, mainly the DEET base, against ticks, especially *A. sculptum*, since the bioassays performed satisfactory repellency. Results were obtained in the following order: Off!^®^ > Repex^®^ > Exposis^®^ > SBP^®^ > Johnson^®^ > Henlau^®^. DEET-based repellents showed the best repellency results against *A. sculptum* in all bioassays carried out, which corroborated Leal [15] who described the compound as the gold standard of repellents with proven efficiency against insect species.

The filter paper bioassay indicated all bases obtained different results from the control, with high percentages of repellency (DEET, Icaridin, and IR3535). It is noteworthy the highest percentages of repellency is obtained with DEET-based repellents: (i) Repelex^®^ (95.42%) and (ii) OFF!^®^ (97.08%). It is noted, according to the information in Table 1, the concentration of the active ingredient in DEET-based repellents is lower, although they obtain the highest percentages of repellency.

In the study conducted by Soares et al. [18], in which the fingertip bioassay was used to analyze the repellency of *A. sculptum*, a repellency rate greater than 90% was observed in all DEET concentrations tested (0.200 to 0.025 mg·cm^2^), in the range between 10 min and 1 h after application, representing a viable alternative against this Ixodidae.

It is also noteworthy, regarding tests with filter paper, DEET repellent also demonstrate effectiveness against the *Amblyomma americanum* tick, in a study carried out by Carroll et al. [20], confirming the use of this base in the prevention of diseases transmitted by these arthropods.

Jesenieu et al. [29], also found satisfactory repellency results against *Amblyomma hebraeum* using DEET-based repellent. Two hours after applying the repellent, a repellency percentage of 89% was achieved. Gomes et al. [30] pointed out commercial repellents based on DEET were the most relevant when it came to repelling arthropods. The present study used products containing the DEET to evaluate the repellency efficiency against *A. sculptum*. The results obtained demonstrate evidence that proved the high performance in the repellency against the star tick.

Meng et al. [31] also obtained a better repellency result with the DEET base in filter paper bioassay. In these studies, the authors evaluated DEET repellency and eight commercial essential oils (oregano, cloves, thyme, vetiver, sandalwood, cinnamon, cedar, and peppermint) against star tick nymphs. The results showed the effective DEET concentration that repelled 50% of the ticks was estimated at 0.02 mg/cm^2^, while that of essential oils was between 0.113 and 0.297 mg/cm^2^. The essential oil of oregano was the most efficient of all the oils tested, but none of the essential oils showed a higher level of repellency than DEET.

Still referring to the filter paper bioassay, Bissinger et al. [32] evaluated the efficiency of the commercial repellent BioUD, which had 2-undecanone as its active ingredient, which was derived from wild tomatoes, compared with DEET. In the study, it was found the BioUD repellent was 7.75% effective, while the DEET-based repellent was 98.11%. The results of this research corroborate the findings of the present study, demonstrating the DEET base has a high potential for repellency efficiency against the star tick.

In relation to the fingertip bioassay, OFF!^®^ repellent, over the five hours of testing, obtained a repellency percentage of 96.67%, compared to 47.7% for Repelex^®^ and 55.5% for Exposis^®^. In a similar study, using fingertip bioassay, Perez et al. [33] also obtained high repellency efficiency against the star tick. In this study, two hours of protection and 98% repellency against unfed *A. sculptum* nymphs were observed using Nexcare repellent at concentrations of 7%, 14%, 25%, and 50%.

It appears investigations into the use of repellents to control ticks are becoming increasingly frequent in recent years, as can be seen in the reviews on the topic by Bissinger, Roe [34], Pages et al. [35], Benelli and Pavela [36]. However, for *A. sculptum*, to date, most repellency evaluation studies have been carried out only under laboratory conditions. The present study gives results that indicate the efficiency of commercial repellents, mainly based on DEET, against the *A. sculptum* tick in field conditions.

Thus, this work filled the gap regarding the possibility of using commercial mosquito repellents against *A. sculptum*, as field experiments were carried out in addition to laboratory tests. Given the efficiency results presented, the use of OFF!^®^ repellent is suggested, as it was the one that significantly repelled ticks the most, compared to the control treatment, in the three bioassays carried out, with 96.08% on filter paper, 96.6% on the fingertip and 100% repellency in the field bioassay.

In this study, DEET products obtained better results in laboratory and field bioassays. Mcmeniman et al. [37] described DEET decreased the sensitivity of olfactory receptor neurons to odors, reducing the responses of neurons stimulated by lactic acid. Furthermore, it also acted as an odorant, altering the host’s chemical profile, preventing host-seeking activity [38,39].

Stefani et al. [40] highlighted those substances exhaled through the skin (lactic acid, sweat, CO_2_, among others) and the presence of eczema can interfere with the effectiveness of repellents. These factors should be evaluated in future studies, during laboratory and field experiments.

This study was limited to analyze the repellent effect of commercial products against nymphs of the *A. sculptum* tick. Therefore, no analysis of the products against larvae and adult ticks was carried out, making it necsessary to evaluate the repellent effect of these products in the different phases of tick life. In this study, we chose to use *A. sculptum* nymphs since they are mainly responsible for the transmission of the bacteria that caused Spotted Fever.

It is important to expand the indication of commercial repellents, especially those based on DEET for ticks, considering they are already registered with ANVISA (National Health Surveillance Agency) and respect the resolution that provides for the technical requirements for granting registration of insect repellent cosmetic products [40]. Furthermore, new repellency studies are suggested for other tick species of public health importance, taking into account the accessibility of these products on the market.

## 5. Conclusions

It is concluded that the repellents tested function as a physical barrier for the movement of *A. sculptum*, allowing high effectiveness in repelling with brand performance in the following order: OFF!^®^ > Repelex^®^ > Exposis^®^. Among the brands tested in the three bioassays carried out, OFF!^®^ repellent was the one that obtained the best result in all bioassays, and may be indicated for use as a repellent against *A. sculptum* tick nymphs.

## Figures and Tables

**Table 1:** Commercial repellents registered in Brazil, for protection against mosquito bites, used in repellency tests against *Amblyomma sculptum* nymphs.

Commercial Name	Way of Applying	Active Principle (Product Concentration)	Registration Number (ANVISA)	Manufacturing Company
1 OFF!^®^ Family	Spray	DEET (6.65%)	201920481	Reckitt Benckiser
2 Repelex^®^ Super	Spray	DEET (6.79%)	203450001	Reckitt Benckiser
3 Exposis^®^ Extreme	Spray	Icaridina (20.00%)	203451022	Reckitt Benckiser
4 SBP^®^ Advanced Family	Spray	Icaridina (9.98%)	03451022	Reckitt Benckiser
5 Johnsons^®^ Baby Loção	Lotion	IR3535 (12.50%)	200920551	Johnson & Johnson
6 Henlau^®^ Repelente	Spray	IR3535 (20.00%)	227430204	Henlau

**Table 2:** Bioassay of filter paper for repellency of the *Amblyomma sculptum* species using commercial repellents OFF!^®^, Repelex, SBP, Exposis, Henlau and Jonhson, in 2019.

Repellent	Total of Nymphs Repelled	Repellency (%)	Mean *	Standard Deviation *	Coefficient of Variance *
Control	101	43.33	2.10 ^a^	1.08	51.17
OFF!^®^	233	97.08	4.50 ^b^	0.50	10.40
Repelex^®^	229	95.42	4.77 ^b^	0.47	9.90
SBP^®^	225	93.33	4.67 ^b^	0.72	15.52
Exposis^®^	227	94.58	4.73 ^b^	0.45	9.50
Henlau^®^	179	75.42	3.77 ^c^	1.13	30.08
Jonhson^®^	183	76.25	3.81 ^c^	1.12	29.47

* The values presented refer to the general analysis of each repetition that used 5 nymphs. ^a, b, c^ Different letters have significant statistical differences using the Kruskal-Wallis test, followed by the Dunn’s test, with *p*-values 0.05.

**Table 3:** Fingertip bioassay for repellency of the species *Amblyomma sculptum* using commercial repellents OFF!^®^, Repelex^®^ and Exposis^®^, in 2020.

Repellent	Total of Nymphs Repelled	Repellency (%)	Mean *	Standard Deviation *	Coefficient of Variance *
OFF!^®^	174	96.6	5.80 ^a^	0.41	7.01
Repelex^®^	86	47.7	2.87 ^b^	2.15	74.84
Exposis	100	55.5	3.33 ^b^	1.79	53.63

* The values presented refer to the general analysis of each repetition that used 5 nymphs. ^a, b^ Different letters have significant statistical differences using the Kruskal-Wallis test, followed by the Dunn’s test, with *p*-values 0.05.

**Table 4:** Field bioassays carried out with the commercial repellents OFF!^®^, Repelex^®^ and Exposis^®^ in areas infested by the species *Amblyomma sculptum* at the School of Agronomy at the Federal University of Goias, Goiania/GO, in June 2021.

Repellent	Total of Nymphs Collected	Repellency (%)	Mean	Standard Deviation	Coefficient of Variance
Control	434	0	12.44 ^a^	4.18	33.60
OFF!^®^	0	100	0.00 ^b^	0.00	-
Repelex^®^	14	96.8	0.38 ^b^	1.37	362.63
Exposis^®^	10	93.1	0.82 ^b^	1.09	132.95

^a, b^ Different letters have significant statistical differences using the Kruskal-Wallis test, followed by the Dunn’s test, with *p*-values 0.05.

## Data Availability

All subjects gave their informed consent for inclusion before they participated in the study. The study was conducted in accordance with the Declaration of Helsinki, and the protocol was approved by the Ethics Committee of no. 4.955.565.
